# Genetic diversity and structure of core collection of winter mushroom (*Flammulina velutipes*) developed by genomic SSR markers

**DOI:** 10.1186/s41065-017-0038-0

**Published:** 2017-07-03

**Authors:** Xiao Bin Liu, Jing Li, Zhu L. Yang

**Affiliations:** 1 Key Laboratory for Plant Diversity and Biogeography of East Asia, Kunming Institute of Botany, Chinese Academy of Sciences, Kunming, Yunnan 650201 China; 2 University of Chinese Academy of Sciences, Beijing, 100049 China; 3 State Key Laboratory of Conservation and Utilization for Bioresources in Yunnan, Yunnan University, Kunming, Yunnan 650091 China

**Keywords:** Core set, Molecular breeding, Molecular marker, Microsatellites

## Abstract

**Background:**

A core collection is a subset of an entire collection that represents as much of the genetic diversity of the entire collection as possible. The establishment of a core collection for crops is practical for efficient management and use of germplasm. However, the establishment of a core collection of mushrooms is still in its infancy, and no established core collection of the economically important species *Flammulina velutipes* has been reported.

**Results:**

We established the first core collection of *F. velutipes*, containing 32 strains based on 81 genetically different *F. veltuipes* strains. The allele retention proportion of the core collection for the entire collection was 100%. Moreover, the genetic diversity parameters (the effective number of alleles, Nei’s expected heterozygosity, the number of observed heterozygosity, and Shannon’s information index) of the core collection showed no significant differences from the entire collection (*p* > 0.01). Thus, the core collection is representative of the genetic diversity of the entire collection. Genetic structure analyses of the core collection revealed that the 32 strains could be clustered into 6 groups, among which groups 1 to 3 were cultivars and groups 4 to 6 were wild strains. The wild strains from different locations harbor their own specific alleles, and were clustered stringently in accordance with their geographic origins. Genetic diversity analyses of the core collection revealed that the wild strains possessed greater genetic diversity than the cultivars.

**Conclusion:**

We established the first core collection of *F. velutipes* in China, which is an important platform for efficient breeding of this mushroom in the future. In addition, the wild strains in the core collection possess favorable agronomic characters and produce unique bioactive compounds, adding value to the platform. More attention should be paid to wild strains in further strain breeding.

**Electronic supplementary material:**

The online version of this article (doi:10.1186/s41065-017-0038-0) contains supplementary material, which is available to authorized users.

## Background

A core collection is a subset of accessions that presents the maximum possible genetic diversity contained in an entire collection with minimum redundancy [1, 2]. The establishment of a core collection for crops is practical for efficient management of germplasm. Core collections of most major food crops, such as *Oryza sativa*, *Zea mays*, *Glycine max* and *Triticum aestivum*, have already been established [3–7].

A core collection is traditionally constructed based on morphological and agronomic characters using different strategies, such as the constant allocation (C) strategy, the logarithm (L) strategy, the proportional allocation (P) strategy, and the random sampling (R) strategy [2, 8–10]. However, most morphological and agronomic characters are quantitative traits that can be easily affected by environmental variation [11–13]. Therefore, phenotypic data cannot directly reflect the genetic diversity of germplasm resources [11].

Conversely, molecular markers can directly reflect a germplasm’s genetic diversity at the DNA sequence level. Compared with other molecular markers, simple sequence repeats (SSRs) are randomly repeated DNA sequences, generally 1 to 6 base pairs in length per unit. SSRs can spread extensively throughout a genome. They are typically co-dominant, highly polymorphic, reproducible and easy to score [14–16]. Based on molecular marker data, Kim et al. [17] developed software named PowerCore by applying the advanced maximization (M) strategy with heuristic searching to establish a core collection (allele mining collection); it allows all alleles to be captured in a minimum number of accessions [17]. It has been successfully used with many economically important crops, such as *Oryza sativa*, *Glycine max*, *Olea europaea*, *Vigna radiata*, and *Sesamum indicum* and has been proven to be most suitable for establishing a core collection based on molecular data [18–22].

However, the development of core collections of edible mushrooms is still at an early stage, and core collections have been established only in *Pleurotus ostreatus* and *Lentinula edodes* [23–25]. *Flammulina velutipes* is cultivated on a large scale in East Asia [26–28]. China is currently the largest producer of *F. velutipes*, with an annual production of 2.4 million tons [29]. In our previous study, we obtained 124 strains (110 cultivars from the spawn market of China and 14 wild strains from Yunnan, Sichuan, and Hunan provinces), and excluded cultivars labeled with confusing names, then screened out 81 strains that are genetically different [30]. In order to efficiently manage and utilize of these genetically different strains, a smaller representative core collection without redundant strains is urgently needed.

In this study, we aimed to (i) establish the core collection of *F. velutipes*; (ii) evaluate the genetic diversity of the core collection and the entire collection; and (iii) analyze the core collection’s genetic structure.

## Methods

### Strain materials and DNA extraction

We used 81 strains of *F. velutipes* in this study, including 67 cultivars and 14 wild strains (Additional file 1: Table S1). Genomic DNA was extracted for each strain with the CTAB-based method [31]. In each case, fresh mycelium was harvested from potato dextrose agar medium after inoculation for 10 days at 23 °C. The DNA concentration and purity were measured with a NanoDrop2000 spectrophotometer. The DNA solution of each sample was diluted to 100 ng/μl.

### SSR genotyping

The 25 polymorphic SSR markers used in this study were developed by our previous study [30]. The forward primer of each SSR was labeled with fluorescent dye (FAM) at the 5′ end (TSINGKE, Kunming). Polymerase chain reactions (PCR) were carried out in a total volume of 25 μl, containing 1 μl template DNA, 1 μl bovine serum albumin, 2.5 μl reaction buffer, 0.5 μl deoxynucleoside triphosphate, 1 μl for each primer, 0.3 μl Taq DNA polymerase, and 17.7 μl ddH_2_O. PCR was conducted on an ABI 2720 Thermal Cycler (Applied Biosystems, Foster City, CA) or an Eppendorf Master Cycler (Netheler-Hinz, Hamburg, Germany) under the following parameters: 94 °C for 4 min, then 35 cycles of 94 °C for 30 s, 55 °C for 30 s, and 72 °C for 30 s, followed by a final extension step of 72 °C for 8 min. The PCR products were run in an ABI 3730 Genetic Analyzer using GeneScan 500 Rox as a size standard (Applied Biosystems); after a denaturation step at 98 °C for 5 min and shock chilling on ice, alleles of each locus were scored in base pairs with the GeneMapper v3.2 software package (Applied Biosystems), the size of the PCR products for each SSR was recorded in an Excel spreadsheet.

### Development of a core collection for *F. velutipes*

The core collection was established based on genotyping data using PowerCore software [17]. The heuristic algorithm that finds the optimum path from the initial to the final stages for sample selection was used (http://genebank.rda).

### Data analysis

The genetic diversity parameters (the effective number of alleles Ae, Nei’s expected heterozygosity H, the number of observed heterozygosity Ho, and Shannon’s information index I) and allele frequency of the core collection and of the entire collection were established with PopGene v1.31 [32]. A dendrogram of the genetic relationships among the core collection strains was constructed based on the simple matching (SM) coefficient by applying the unweighted pair group method with arithmetic mean (UPGMA) using the NTSYSpc v2.10e [33]. The genetic structure of the core collection was analyzed with STRUCTURE v2.3.4 based on an admixture model. Models were tested for K-values ranging from 2 to 10, with 10 independent runs per K value. For each run, the initial burn-in period was set to 100,000 with 100,000 MCMC iterations. To determine the most probable value of K, the deltaK method was used and implemented in Structure Harvester [34, 35].

## Results

### Core collection construction

A total of 153 alleles were amplified by 25 SSRs in the 81 strains [30]. In this study, based on PowerCore calculation, the 153 alleles could be represented using a minimum of 32 strains, including 19 cultivars and 13 wild strains (Table 1). This finding suggests that the 32 strains could be a core collection of the 81 strains. The core collection sampling proportion is about 39.5%: for cultivars about 27.9%, and for wild strains about 92.9%.

**Table 1:** Strains in the core collection selected by PowerCore

Strain	Cultivar/wild	Pileus color	origin	Strain	Cultivar/wild	Pileus color	origin
F37	Cultivar	Yellow	Shandong	F80	Cultivar	Yellow	Henan
F114	Cultivar	Yellow	Shandong	F87	Cultivar	Yellow	Hebei
F1	Cultivar	Yellow	Beijing	F79	Cultivar	Yellow	Henan
F6	Cultivar	Yellow	Beijing	F146	Wild	Yellow	Hunan
F89	Cultivar	Yellow	Hebei	F147	Wild	Yellow	Hunan
F115	Cultivar	Yellow	Shandong	F148	Wild	Yellow	Hunan
F58	Cultivar	Yellow	Heilongjiang	F77	Wild	Yellow	Jilin
F112	Cultivar	Yellow	Shandong	F92	Wild	Yellow	Sichuan
F117	Cultivar	White	Liaoning	F93	Wild	Yellow	Sichuan
F106	Cultivar	Yellow	Fujian	F94	Wild	Yellow	Sichuan
F133	Cultivar	White	Hunan	F91	Wild	Yellow	Yunnan
F19	Cultivar	White	Sichuan	F149	Wild	Yellow	Yunnan
F78	Cultivar	White	Jilin	F99	Wild	Yellow	Yunnan
F26	Cultivar	White	Shijiazhuang	F101	Wild	Yellow	Yunnan
F116	Cultivar	White	Liaoning	F98	Wild	Yellow	Yunnan
F151	Cultivar	White	Kunming	F103	Wild	Yellow	Yunnan

### Genetic diversity of the core collection

**Fig. 1:**
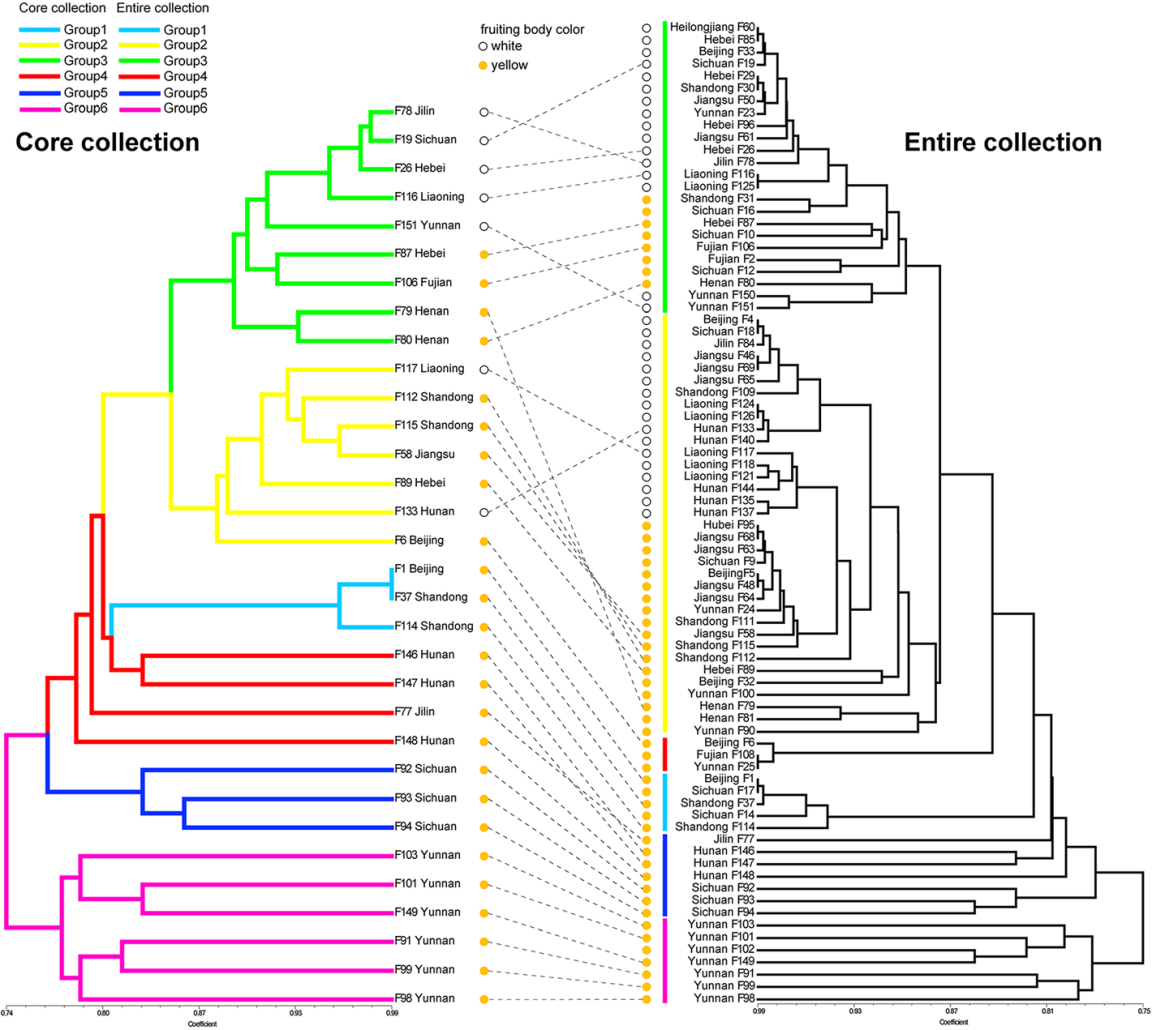
UPGMA dendrogram of core collection (32 accessions) and entire collection (81 accessions) of *F. velutipes* constructed based on genetic similarity coefficients of SSR data in the core collection and entire collection, respectively. Strains connected with dashed lines show placement of core collection strains in entire collection

Statistics to describe the genetic diversity of the core collection and the entire collection for 25 SSR markers are summarized in Table 2. The *t*-tests of the mean genetic diversity parameters (Ae, H, Ho, I) of the core collection and the entire collection were non-significant (*p* > 0.01) (Table 2), which reveals that the genetic diversity of the core collection has no significant difference from that of the entire collection. In addition, based on an UPGMA dendrogram of the core collection and the entire collection (Fig. 1), the strains in different groups of the entire collection were uniformly selected for the core collection. Thus, the core collection could represent the genetic diversity of the entire collection. However, the distributions of allele frequency differ between the entire collection and the core collection (Additional file 2: Table S2).

**Table 2:** Genetic diversity parameters in entire collection and core collection

Locus name	Ae		H		Ho		I	
	Entire	Core	Entire	Core	Entire	Core	Entire	Core
SSR1	1.833	2.779	0.455	0.64	0.37	0.5	0.926	1.29
SSR2	2.846	3.85	0.649	0.74	0.617	0.719	1.286	1.589
SSR4	1.051	1.136	0.049	0.12	0.037	0.094	0.141	0.299
SSR5	1.025	1.065	0.025	0.061	0.025	0.063	0.075	0.161
SSR7	2.949	3.717	0.661	0.731	0.716	0.625	1.241	1.424
SSR15	1.964	1.882	0.491	0.469	0.346	0.25	0.684	0.662
SSR19	1.839	2.004	0.456	0.501	0.58	0.594	0.728	0.854
SSR21	3.905	5.02	0.744	0.801	0.457	0.438	1.576	1.757
SSR22	3.619	5.171	0.724	0.807	0.444	0.313	1.59	1.949
SSR23	1.552	1.468	0.356	0.319	0.37	0.25	0.624	0.618
SSR24	1.613	1.779	0.38	0.438	0.173	0.219	0.78	0.946
SSR25	1.212	1.556	0.175	0.357	0.074	0.125	0.475	0.874
SSR26	1.148	1.253	0.129	0.202	0.136	0.219	0.3	0.45
SSR32	3.187	5.988	0.686	0.833	0.259	0.219	1.669	2.163
SSR45	2.385	3.127	0.581	0.68	0.482	0.313	1.12	1.388
SSR87	2.617	3.894	0.618	0.743	0.457	0.406	1.196	1.559
SSR65	1.276	1.767	0.216	0.434	0.148	0.313	0.536	0.95
SSR124	2.383	2.922	0.580	0.658	0.482	0.563	1.039	1.289
SSR133	2.176	4.047	0.541	0.753	0.222	0.313	1.254	1.755
SSR95	1.895	1.979	0.472	0.495	0.605	0.656	0.725	0.742
SSR107	1.553	2.186	0.356	0.543	0.272	0.344	0.826	1.2
SSR119	1.225	1.535	0.184	0.349	0.086	0.125	0.425	0.726
SSR128	1.705	1.636	0.413	0.389	0.469	0.313	0.647	0.657
SSR132	3.024	4	0.669	0.75	0.667	0.594	1.293	1.521
SSR136	1.119	1.334	0.106	0.251	0.012	0.031	0.25	0.496
mean	2.044	2.684	0.429	0.522	0.34	0.344	0.856	1.093

We further investigated the genetic diversity parameters (Ae, H, Ho, I) of the cultivars and wild strains in core collection, which demonstrated that the wild strains possess greater genetic diversity than the cultivars (Table 3).

**Table 3:** Genetic diversity parameters in cultivars and wild strains of core collection

Locus name	Ae		H		Ho		I	
	Cultivar	Wild	Cultivar	Wild	Cultivar	Wild	Cultivar	Wild
SSR1	1.925	4.225	0.481	0.763	0.421	0.615	0.884	1.554
SSR2	2.704	4.173	0.63	0.76	0.684	0.769	1.156	1.724
SSR4	1.113	1.17	0.101	0.145	0.053	0.154	0.243	0.325
SSR5	1.054	1.08	0.051	0.074	0.053	0.077	0.122	0.163
SSR7	2.854	4.173	0.65	0.76	0.684	0.539	1.169	1.512
SSR15	1.915	1.08	0.478	0.074	0.368	0.077	0.671	0.163
SSR19	1.761	2.414	0.432	0.586	0.632	0.539	0.624	1.077
SSR21	4.349	4.568	0.77	0.781	0.474	0.385	1.68	1.668
SSR22	3.39	5.541	0.705	0.82	0.316	0.308	1.367	1.938
SSR23	1.447	1.49	0.309	0.328	0.368	0.077	0.553	0.619
SSR24	1.296	2.561	0.229	0.61	0.053	0.462	0.389	1.197
SSR25	1.113	2.661	0.101	0.624	0.105	0.154	0.243	1.323
SSR26	1.238	1.266	0.193	0.21	0.211	0.231	0.396	0.431
SSR32	2.935	9.389	0.659	0.894	0.211	0.231	1.313	2.322
SSR45	2.208	4.447	0.547	0.775	0.263	0.385	0.922	1.612
SSR87	2.057	4.225	0.514	0.763	0.368	0.462	0.826	1.693
SSR65	1.054	3.25	0.051	0.692	0.053	0.692	0.122	1.434
SSR124	2.13	2.397	0.531	0.583	0.526	0.615	0.919	1.232
SSR133	2.087	7.042	0.521	0.858	0.158	0.539	0.988	2.018
SSR95	2.034	1.9	0.508	0.473	0.684	0.615	0.779	0.667
SSR107	1.576	3.189	0.366	0.686	0.421	0.231	0.829	1.334
SSR119	1.232	1.988	0.188	0.497	0	0.308	0.337	0.882
SSR128	1.498	1.857	0.332	0.462	0.316	0.308	0.515	0.79
SSR132	3.181	3.634	0.686	0.725	0.684	0.462	1.324	1.391
SSR136	1.111	1.699	0.1	0.411	0	0.077	0.206	0.693
mean	1.97	3.257**	0.405	0.574*	0.324	0.372*	0.743	1.19**

*indicates significant difference in genetic diversity parameter at 0.05 level between the cultivars and wild strains

**indicates significant difference in genetic diversity parameter at 0.01 level between the cultivars and wild strains

**Fig. 2:**
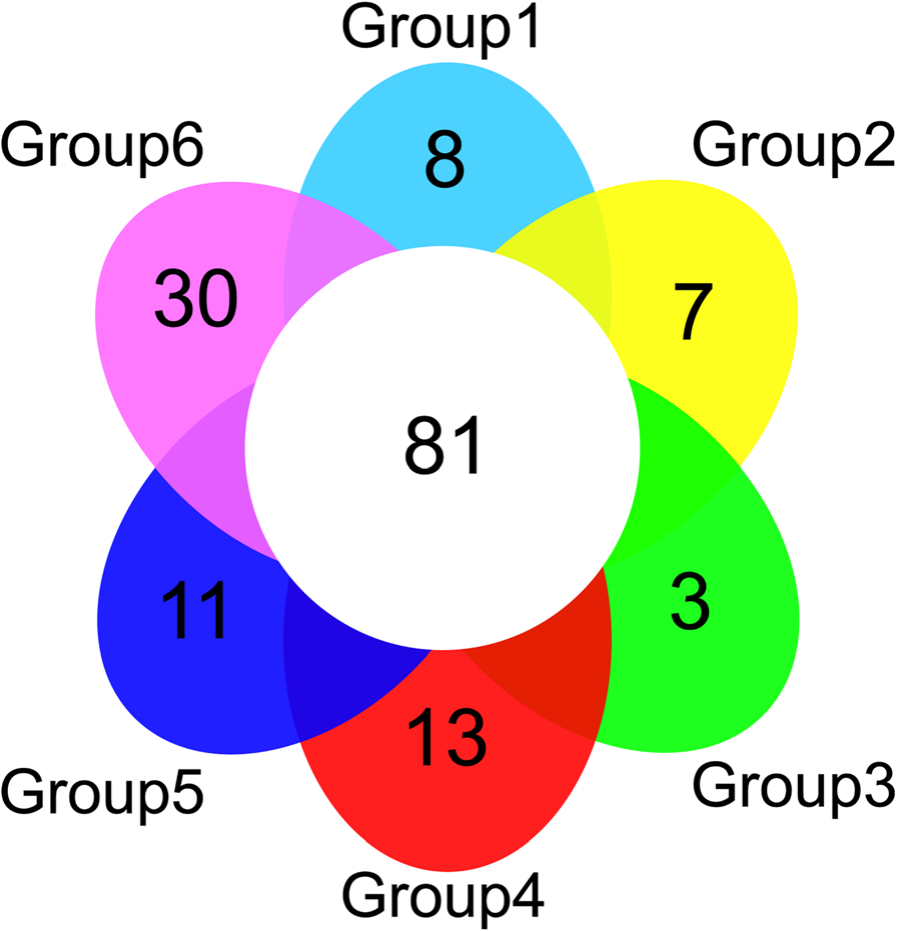
Venn diagram demonstrates common and specific alleles distributed in 6 groups

Among the 153 alleles in the core collection, 72 (47%) were specific for each group and could differentiate the six groups from each other (Fig. 2). Group 6 had the highest number of specific alleles (30), followed by group 4 (13), group 5 (11), group 1 (8), group 2 (7), and group 3 (3) (Fig. 2). Nearly 53% of the alleles (81 of 153) were common to all the groups and can thus be categorized as conserved alleles (Fig. 2).

### Genetic structure of the core collection

**Fig. 3:**
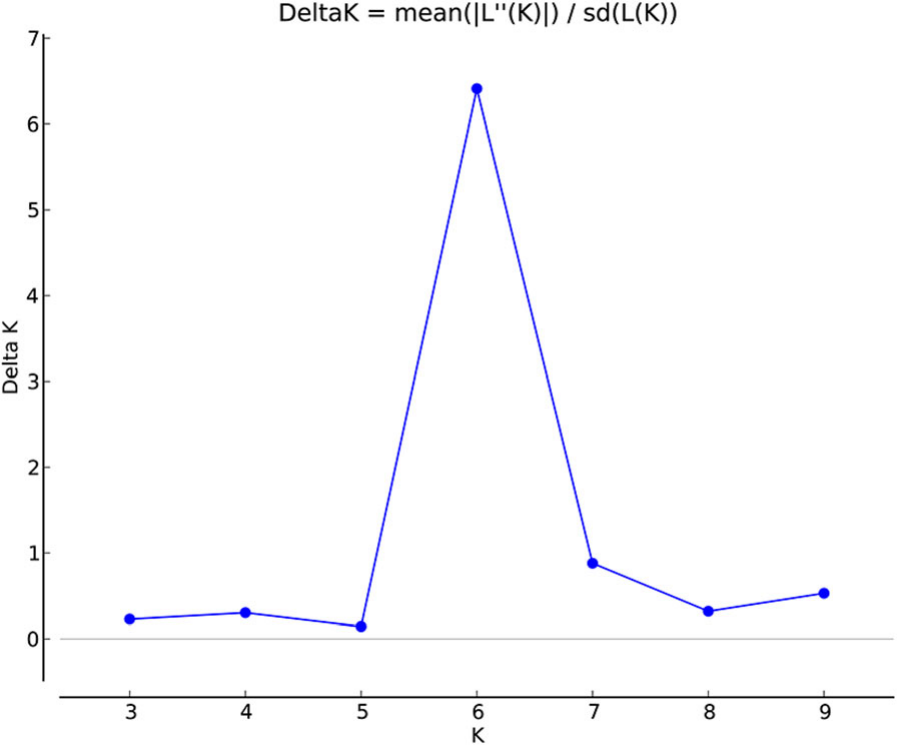
Estimation of number of populations for K ranging from 2 to 10 by ΔK values

**Fig. 4:**
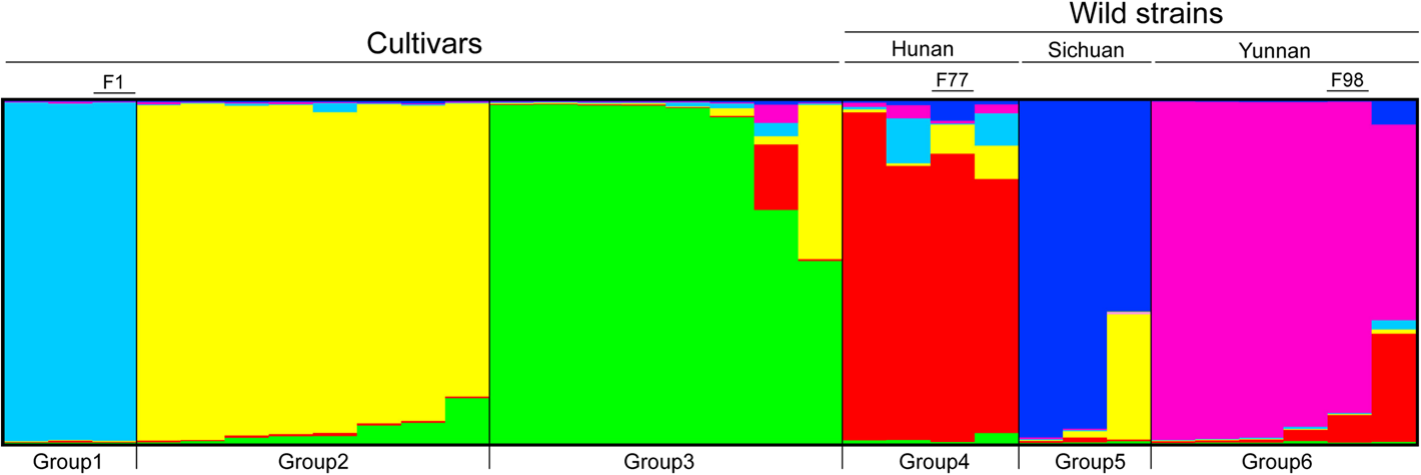
Estimated genetic structure for K = 6. Each group is labeled beneath the figure. Cultivars and wild strains are labeled above the figure. Each bar represents a single individual

The admixture model-based clustering method was used in the STRUCTURE program to infer the genetic structure of the core collection. The optimum number of K was analyzed using delta K (ΔK). A strong peak of ΔK is six, which indicated that there were six groups in the core collection (Fig. 3). The cultivars were assigned to groups 1 to 3, and the wild strains were assigned to groups 4 to 6 (Fig. 4).

A similar result was also shown in the dendrogram constructed with the UPGMA method (Fig. 1). For the cultivars, white strains were assigned to groups 2 and 3, and yellow strains were distributed throughout groups 1 to 3. The wild strains were clustered in groups 4 to 6, and each group was stringently in accordance with its geographic origins. The strains in group 4 were collected from Hunan Province, with the exception of strain F77, which was purchased from Spawn Company in Jilin Province in northeastern China. This strain shares similar alleles with the strains collected from Hunan Province, indicating that it may have been isolated from Hunan Province. The strains in groups 5 and 6 were collected from Sichuan and Yunnan Provinces, respectively.

## Discussion

### Representation of the core collection

The successful formation of a core collection depends on maximum allelic representation efficiency and elimination of redundancy from the entire collection [2]. In this study, we successfully developed a core collection of *F. velutipes* with 100% allelic representation under 39.5% of the sampling proportion (28.4% for cultivars and 92.9% for wild strains) based on 25 SSR markers. The genetic diversity parameters of the core collection could represent of the entire collection. And the strains selected in the core collection can represent the different allele components of each group in the entire collection (Fig. 1). Our results proved that the advanced M strategy is powerful in capturing 100% allelic diversity in a core collection [17, 22]. The differences of the allele frequency between the entire collection and the core collection may be due to the redundant alleles including some homozygous loci that were excluded during the core collection construction.

### Further strain improvement of *F. velutipes* based on the core collection

Most crops inevitably undergo a drastic loss of genetic diversity during cultivation, and *F. velutipes* is no exception [30, 36–38]. Lower genetic diversity among cultivars may lead to inbreeding depression [39]. Thus, the core collection established in this study could effectively protect the cultivars’ heterogeneous germplasm resources and help avoid inbreeding depression for further strain improvement. Furthermore, the specific alleles harbored in different groups of cultivars may indicate different agronomic characters in each group. For example, in our previous experiment, the mycelium growth rate at 23 °C of a yellow strain F1 (6.38 ± 0.1 mm/d) in group 1 was significantly greater than that of the industrialized white strain F3 (6.35 ± 0.07 mm/d) (*p* < 0.01) (unpublished data). Therefore, F1 could be used to crossbreed with the industrialized white strains to facilitate mycelium growth and shorten the production time.

The wild strains harbor higher genetic diversity than the cultivars in the core collection. Meanwhile, several economically important agronomic traits, such as tolerance to high temperature and rich contents of sesquiterpenes, can also be found in wild strains [30, 40]. In the cultivation of *F. velutipes*, temperature is usually an important limiting factor. The cultivars’ fruiting temperature needed for stringent control is less than 15 °C, which will result in high energy costs [30]. However, we have gathered several wild strains of *F. velutipes* from subtropical regions of China in summer, despite most of the wild strains of this species mainly forming fruiting bodies in winter. Thus, those wild strains are ideal samples to domesticate for tolerance to high temperatures. In fact, we did find a wild strain (F98), collected from Longling, Yunnan, that can grow more vigorously than cultivars at a higher temperature (18 °C) [30]. Further analyses on chemical components showed that this strain contains 15 new sesquiterpenes with various skeletons, some of which showed moderate antidiabetes and antitumor bioactivity [40]. Thus, it is quite essential to keep as many wild strains as possible in constructing a core collection for *F. velutipes*.

## Conclusions

In conclusion, we have established the first core collection of *F. velutipes* in China, which is an important platform for efficient breeding of this mushroom in the future. The core collection is representative of the entire collection. In addition, the wild strains in the core collection possess favorable agronomic characters and produce unique bioactive compounds, adding value to the platform. More attention should be paid to wild strains in further strain breeding.

## Additional files

Additional file 1: Strains used in this study. (DOC 82 kb)

Additional file 2: Allele frequence distributions between entire collection and core collection. (XLS 46 kb)
