# Supplementary material for: Genetic diversity and structure of core collection of winter mushroom (Flammulina velutipes) developed by genomic SSR markers
Source: Hereditas. 2017 Jul 3;155:3. doi: 10.1186/s41065-017-0038-0 (PMC5496253; doi:10.1186/s41065-017-0038-0)
Supplement: Supplementary file 1 — Strains used in this study. (DOC 82 kb) [file 41065_2017_38_MOESM1_ESM.doc]

**Supplementary table 1** Strains used in this study

| Code | Origin | Cultivar/Wild |
| --- | --- | --- |
| F1 | Beijing | cultivar |
| F2 | Beijing | cultivar |
| F5 | Beijing | cultivar |
| F6 | Beijing | cultivar |
| F9 | Sichuan | cultivar |
| F10 | Sichuan | cultivar |
| F12 | Sichuan | cultivar |
| F14 | Sichuan | cultivar |
| F16 | Sichuan | cultivar |
| F17 | Sichuan | cultivar |
| F18 | Sichuan | cultivar |
| F19 | Sichuan | cultivar |
| F23 | Yunnan | cultivar |
| F24 | Yunnan | cultivar |
| F25 | Yunnan | cultivar |
| F26 | Hebei | cultivar |
| F29 | Hebei | cultivar |
| F30 | Shandong | cultivar |
| F31 | Shandong | cultivar |
| F32 | Beijing | cultivar |
| F33 | Beijing | cultivar |
| F37 | Shandong | cultivar |
| F46 | Jiangsu | cultivar |
| F48 | Jiangsu | cultivar |
| F50 | Jiangsu | cultivar |
| F58 | Jiangsu | cultivar |
| F60 | Heilongjiang | cultivar |
| F61 | Jiangsu | cultivar |
| F63 | Jiangsu | cultivar |
| F64 | Jiangsu | cultivar |
| F65 | Jiangsu | cultivar |
| F68 | Jiangsu | cultivar |
| F69 | Jiangsu | cultivar |
| F77 | Jilin | wild |
| F78 | Jilin | cultivar |
| F79 | Henan | cultivar |
| F80 | Henan | cultivar |
| F81 | Henan | cultivar |
| F82 | Jilin | cultivar |
| F84 | Jilin | cultivar |
| F85 | Hebei | cultivar |
| F87 | Hebei | cultivar |
| F89 | Hebei | cultivar |
| F90 | Yunnan | cultivar |
| F91 | Yunnan | wild |
| F92 | Sichuan | wild |
| F93 | Sichuan | wild |
| F94 | Sichuan | wild |
| F95 | Hebei | cultivar |
| F96 | Hebei | cultivar |
| F98 | Yunnan | wild |
| F99 | Yunnan | wild |
| F100 | Yunnan | cultivar |
| F101 | Yunnan | wild |
| F102 | Yunnan | wild |
| F103 | Yunnan | wild |
| F106 | Fujian | cultivar |
| F108 | Fujian | cultivar |
| F109 | Shandong | cultivar |
| F111 | Shandong | cultivar |
| F112 | Shandong | cultivar |
| F114 | Shandong | cultivar |
| F115 | Shandong | cultivar |
| F116 | Liaoning | cultivar |
| F117 | Liaoning | cultivar |
| F118 | Liaoning | cultivar |
| F121 | Liaoning | cultivar |
| F124 | Liaoning | cultivar |
| F125 | Liaoning | cultivar |
| F126 | Liaoning | cultivar |
| F133 | Hunan | cultivar |
| F135 | Hunan | cultivar |
| F137 | Hunan | cultivar |
| F140 | Hunan | cultivar |
| F144 | Hunan | cultivar |
| F146 | Hunan | wild |
| F147 | Hunan | wild |
| F148 | Hunan | wild |
| F149 | Yunnan | wild |
| F150 | Yunnan | cultivar |
| F151 | Yunnan | cultivar |
